# Immunohistochemical Localization of Fibroblast Activation Protein in Coronary Arteries with Different Forms of Atherosclerosis

**DOI:** 10.3390/metabo14110573

**Published:** 2024-10-25

**Authors:** Adam Mohmand-Borkowski, Tomasz Rozmyslowicz

**Affiliations:** 1 Department of Cardiology, Cape Cod Hospital, Hyannis, MA 02601, USA; 2 Department of Pathology and Laboratory Medicine, University of Pennsylvania, Philadelphia, PA 19104, USA; rozmyslo@pennmedicine.upenn.edu

**Keywords:** fibroblast activation protein, vascular smooth muscle cells, atherosclerotic plaque

## Abstract

**Background:** Fibroblast activation protein (FAP) is a cell surface glycoprotein expressed by myofibroblasts in areas of active tissue remodeling. It plays a potentially important role in cardiac remodeling, atherosclerotic plaque formation, and plaque rupture. Given the distinct pathophysiology and morphology of different forms of atherosclerosis, we analyzed FAP expression in human coronary vessels with no coronary artery disease, atherosclerotic plaques at different levels of progression, and other distinct forms of coronary disease in post bypass vein grafting and cardiac allograft vasculopathy after a heart transplant. **Methods:** Immunohistochemical staining with monoclonal F19 mouse anti-human FAP antibody was performed to identify FAP in human atherosclerotic plaques, coronary bypass atherosclerosis, and post-transplant arteriosclerosis. The presence and distribution of FAP in different types and stages of human atherosclerosis were compared. **Results:** There was no FAP staining in patients with no significant coronary disease. All different types of human atherosclerotic lesioning lesions showed the presence of FAP expression, with different staining patterns in advanced atherosclerotic plaque, vein graft atherosclerosis lesions, and arteriosclerosis after a heart transplant. **Conclusions:** These data suggest that FAP may be a potential diagnostic marker and target for interventions, not only in coronary atherosclerotic plaque, but also in other forms of coronary disease, which have distinct pathophysiologies and currently limited treatment options.

## 1. Introduction

Extracellular matrix (ECM) turnover is critical for atherosclerotic plaque progression, resistance to rupture, and resultant acute coronary syndromes. Vascular smooth muscle cell (VSMC) activation plays a key role in this process and other forms of coronary atherosclerosis, such as restenosis post percutaneous interventions, post bypass accelerated atherosclerosis, and allopathy after a heart transplant. A better understanding of VSMC and myofibroblast proliferation and signaling in the different forms of atherosclerosis is necessary before developing future therapies to prevent acute coronary syndromes [1,2,3,4,5,6,7,8] and therapies for bypassing accelerated atherosclerosis and allopathy after a heart transplant.

Fibroblast activation protein (FAP) is a cell surface glycoprotein expressed by myofibroblastic cells in areas of active tissue remodeling. It has dipeptidyl peptidase and collagenase activity, leading to ECM degradation [9,10,11,12] and the loss of FAP-expression-upregulated compensatory pathways such as metalloproteinase activation [13]. As FAP is uniquely present in chronic inflammatory lesions and has an important role in ECM turnover, it appears to have characteristics that play a role in atherosclerosis and atherosclerotic plaque rupture. A study that focused on the presence of FAP in human coronary plaque suggested increased expression of FAP in thin-cap versus thick-cap human atherosclerotic plaques [14]. The presence of FAP in advanced coronary lesions has previously been shown [15]. However, a study on human carotid atherosclerotic plaques showed only a moderately higher level of FAP expression in atherosclerosis plaques than in normal arteries with the binding of a boronic acid-based FAP inhibitor, and this expression was independent of plaque vulnerability [16]. It has been shown that after acute myocardial infarction, FAP upregulation occurs in the infarct region and attached healthy myocardium [17], suggesting that FAP may play a role not only in the replacement but also in reactive fibrosis, which occurs in other forms of atherosclerosis. Given the multifactorial and not fully understood mechanisms of cardiac allograft vasculopathy and post bypass atherosclerosis, specific therapeutic targets are lacking. The main goal of this study was to analyze the expression and localization of FAP in different types of atherosclerotic lesions: human atherosclerotic plaques, atherosclerosis postbypass surgery, and after a heart transplant.

## 2. Materials and Methods

Coronary artery sections were obtained from patients with dilated cardiomyopathy without atherosclerosis and patients with coronary artery disease undergoing heart transplantation. Postmortem coronary sections from patients with prior coronary artery bypass grafting and allopathy after heart transplant were used to analyze bypass atherosclerosis and post-transplant atherosclerosis. Twenty sections in each group were used in the experiment. With the exception of bypass vessels, all other sections of coronary lesions were examined from left anterior descending arteries to limit coronary vessel variations. Atherosclerotic lesions were classified into three histological categories: mild, moderate, and advanced. Tissue with coronary sections was fixed with 4% formalin and embedded into paraffin.

Anti-FAP antibodies were produced and purified in the following manner: F19 hybrid cells producing anti-human FAP were grown in a suspension, and once they reached 80–100 million cells, they were transferred to an Integra CL flask, and crude anti-human FAP IgG1 was obtained. Antibodies were precipitated using ammonium sulfate and then purified using a protein G sepharose column with Western Blot used to confirm the predicted molecular weight. The monoclonal anti-FAP antibody was labeled with fluorescein isothiocyanate (FITC) so that it could be observed directly with flow cytometry.

Immunohistochemical staining was performed to identify FAP in human atherosclerotic plaques, coronary bypass atherosclerosis, and post-transplant arteriosclerosis. Each frozen paraffin block was cut into 5 µm thick sections, deparaffinized, rehydrated, incubated in 0.3% H_2_O_2_ for 30 min to block endogenous peroxidases, and washed with PBS buffer. The following antibodies were used in the experiment: mouse anti-human IgG1 (F19) with appropriate isotype match control as the primary antibody and then biotin-labeled goat anti-mouse antibody (Sigma-Aldrich, St Louis, MO, USA) as a secondary antibody. The primary antibody (F19) or matched isotype was applied for staining at a concentration of 10 μg/mL at room temperature for 60 min. Sections were then incubated with a secondary biotin-labeled goat anti-mouse antibody used at 1:200 dilutions, washed, and incubated with a diaminobenzidine (DAB, Sigma-Aldrich, St Louis, MO, USA) reagent for microscopic visualization. The imaging of histological sections was performed using an Olympus BH-2 microscope with an Olympus DP-10 camera (Olympus, Tokyo, Japan).

The presence and distribution of FAP in different types and stages of human atherosclerosis were compared.

## 3. Results

### 3.1. The FAP Expression in Human Coronaries Without and with Coronary Plaques with Different Levels of Atherosclerosis Progression Was Examined. FAP Staining Was Absent in Cross-Sections from Coronary Vessels with No Atherosclerosis, Whereas a Gradual Increase in Staining Was Observed with the Progression of Atherosclerotic Plaques

#### 3.1.1. FAP Staining in Coronary Vessels with No Atherosclerosis and with Coronary Artery Disease

**Figure 1:**
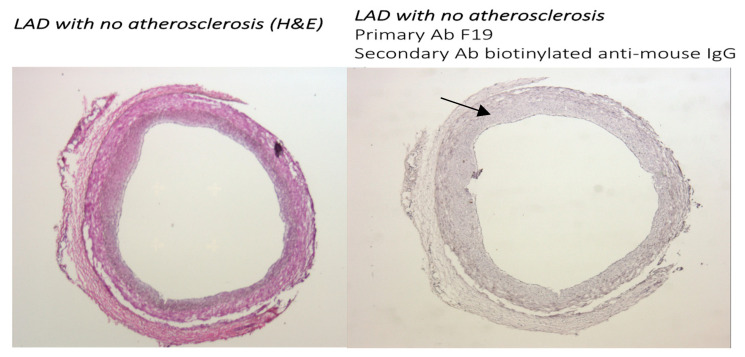
Sections from human coronary artery (5× magnification) from patient with dilated cardiomyopathy with no atherosclerotic plaque after H&E staining (**left**). Staining with F19 mouse anti-human FAP antibody as primary antibody and biotinylated anti-mouse IgG as secondary antibody (**right**). Section from coronary artery with no atherosclerosis shows no staining with F19 (black arrow).

**Figure 2:**
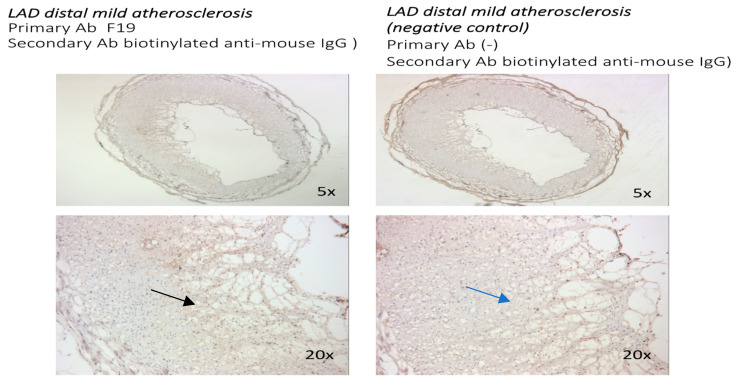
Sections from human coronary artery with mild atherosclerosis (5× and 20× magnifications). Staining with F19 mouse anti-human FAP antibody as primary antibody and biotinylated anti-mouse IgG as secondary antibody (**left**). Negative control with staining with biotinylated anti-mouse IgG (**right**). Weak F19 staining in coronary lesions with mild atherosclerosis (black arrow) compared with negative control (blue arrow).

**Figure 3:**
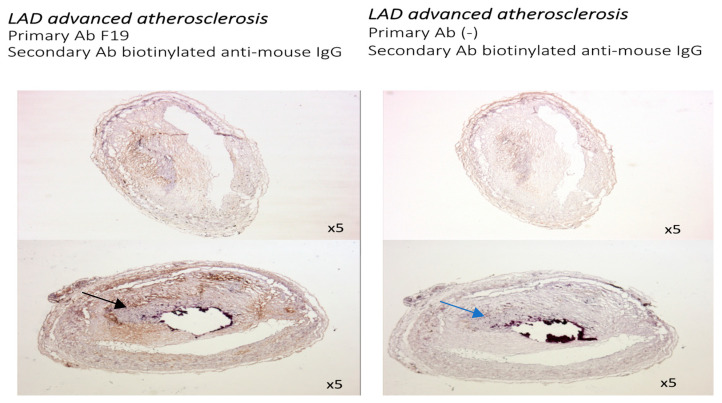
Sections from human coronary artery with severe atherosclerotic plaque (5× magnification). Staining with F19 mouse anti-human FAP antibody as primary antibody and biotinylated anti-mouse IgG as secondary antibody (**left**). Negative control with staining with biotinylated anti-mouse IgG (**right**). Strongly positive F19 staining in advanced atherosclerotic plaque (black arrow) compared with no F19 staining in negative control (blue arrow).

Immunochemistry with the F19 antibody (mouse anti-human FAP) was performed in human coronaries from patients with no significant coronary disease (CAD), as well as mild, moderate, and advanced CAD in the left anterior descending artery. The F19 staining showed no FAP staining in the left anterior descending artery in patients with no significant CAD (Figure 1), faint staining in mild left anterior descending artery lesions (Figure 2), and marked staining in advanced atherosclerotic plaques (Figure 3).

#### 3.1.2. Localization of FAP Staining in Advanced Atherosclerotic Plaques

**Figure 4:**
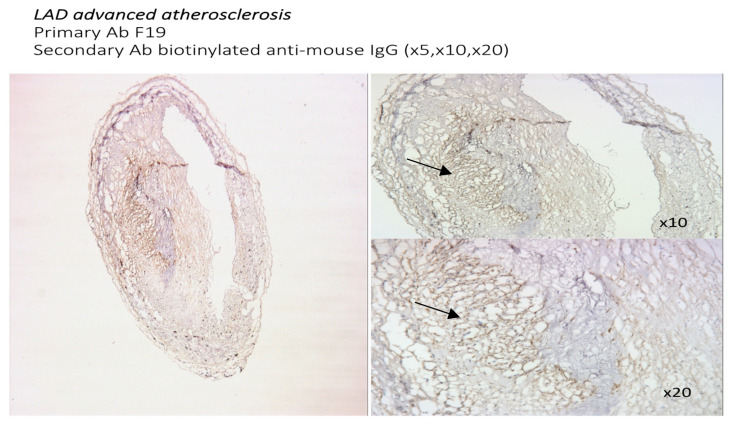
Sections from a human coronary artery with severe atherosclerotic plaque (5×, 10×, and 20× magnification). Staining with an F19 mouse anti-human FAP antibody as the primary antibody and biotinylated anti-mouse IgG as the secondary antibody. Strongly positive F19 staining is localized in the core of the advanced atherosclerotic plaque (black arrow).

In advanced atherosclerotic plaques, FAP was expressed mostly in the atherosclerotic core, with relative sparing of the fibrous cap. Higher-magnification images are presented (Figure 2 and Figure 4) to better compare the FAP staining distribution in early atherosclerosis plaques (20× magnification) and the FAP distribution in advanced atherosclerotic lesions (10× and 20×).

### 3.2. The Presence of FAP Was Also Examined in Other Distinct Models of Atherosclerosis: The Sections of Coronaries in Post Bypass Atherosclerosis (Figure 5) and Cardiac Allograph Vasculopathy (Figure 6) Both Showed Positive and Distinct FAP Staining Compared with Native Coronary Artery Disease

#### 3.2.1. The Presence and Localization of FAP Staining in Postbypass Coronary Atherosclerosis and Heart Transplant Arteriosclerosis

**Figure 5:**
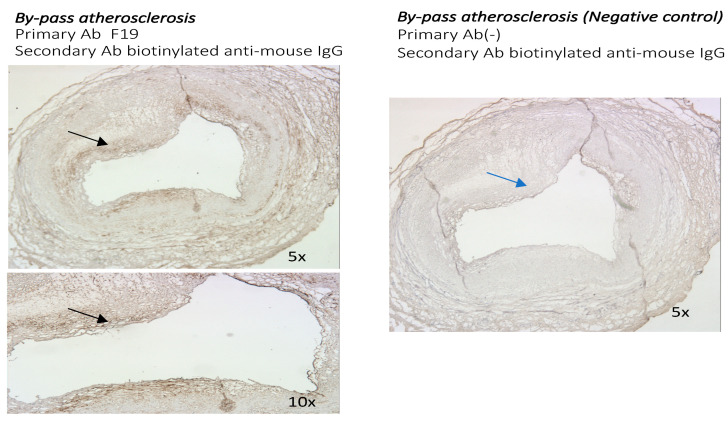
Sections from post bypass atherosclerosis (5× and 10× magnifications). Staining with F19 mouse anti-human FAP antibody as primary antibody and biotinylated anti-mouse IgG as secondary antibody (**left**). Negative control with staining with biotinylated anti-mouse IgG (**right**). Diffuse pattern of F19 staining in sections from post bypass atherosclerosis (black arrow) compared with negative control (blue arrow).

**Figure 6:**
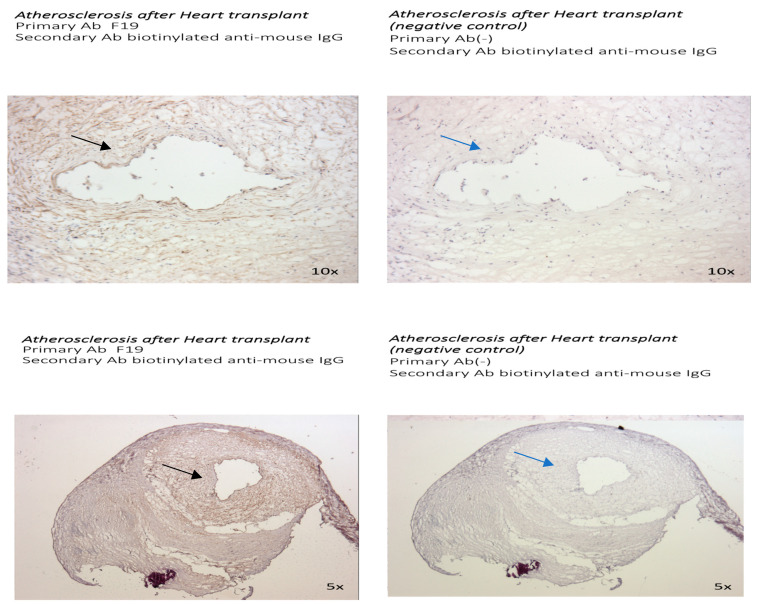
Sections from post-heart transplant arteriosclerosis (5× and 10× magnification). Staining with F19 mouse anti-human FAP antibody as primary antibody and biotinylated anti-mouse IgG as secondary antibody (**left**). Negative control with staining with biotinylated anti-mouse IgG (**right**). Strongly positive diffuse pattern of F19 staining in sections from post-heart transplant arteriosclerosis (black arrow) compared with negative control (blue arrow).

Coronary sections with moderate postbypass coronary atherosclerosis (Figure 5) and with heart transplant arteriosclerosis (Figure 6) showed positive and diffuse patterns of intraluminal FAP staining with the F19 antibody. The FAP staining was present mostly in the tunica intima in vein graft atherosclerosis, whereas a strongly positive diffuse pattern of F19 staining in the tunica intima and media was present in heart transplant arteriosclerosis.

#### 3.2.2. Comparison of FAP Staining in Native Coronary Atherosclerosis with Other Forms of Coronary Artery Disease

With all the different types of human atherosclerotic lesions showing FAP-positive staining, the highest expression with a distinct pattern of staining was found in advanced atherosclerotic plaques, vein graft arteriosclerosis lesions, and atherosclerosis after a heart transplant.

**Figure 7:**
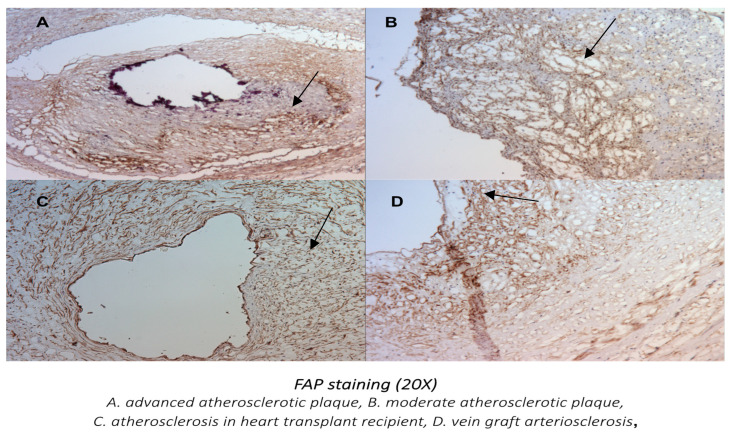
Comparison of the F19 (anti-human FAP Ab) staining of advanced and moderate native coronary atherosclerotic plague, vein graft arteriosclerosis lesion, and post-heart transplant arteriosclerosis (20× magnification). (**A**,**B**) The localization of the most prominent F19 staining in the coronary plaques in native coronary disease (black arrows). (**C**,**D**) The strongly positive diffuse pattern of F19 staining in the tunica intima and media of the heart transplant arteriosclerosis (black arrow) and diffuse pattern of F19 staining in the tunica intima of the vein graft atherosclerosis (black arrow).

Using 20× magnifications of the coronary sections (Figure 7), a more diffuse pattern of FAP staining was visualized in the tunica intima with post bypass atherosclerosis and the tunica intima and media in cardiac allopathy after heart transplant. The most pronounced FAP staining in advanced atherosclerotic lesions was localized in the core of the atherosclerotic plaque.

## 4. Discussion

VSMC migration and proliferation associated with the inflammatory process play major roles in different forms of atherogenesis and acute coronary syndromes [18,19,20,21]. However, the exact role of VSMCs varies throughout different types of atherogenesis and is not fully understood. Once activated, VSMCs may undergo complex changes with different resulting phenotypes, playing a role in different forms and stages of atherosclerosis. The distinct role of activated smooth muscle cells is postulated in different forms of atherosclerosis—native CAD, accelerated atherosclerosis post revascularization, and premature arteriosclerosis—thus contributing to vasospasm and sudden cardiac death in young adults.

In native atherosclerosis, VSMCs play a major role at all stages of plaque formation and in plaque rupture [22,23,24]. Vulnerable plaque hypotheses assume that the “contractile” VSMCs, which are recruited from the media, undergo phenotypic changes to form “synthetic” VSMCs, stimulating the ECM to form the fibrous cap and stabilizing plaques. In addition, growing evidence suggests that VSMCs are more plastic and can exhibit alternative phenotypes, which can affect atherosclerosis progression and plaque rupture both positively and negatively [25,26,27]. The concept of targeting antigens that are selectively expressed on the surface of different subpopulations of myofibroblasts has emerged as a promising strategy for immune-based approaches in cancer therapy and experimental treatments for CAD. The identification of targets and development of selective inhibitors could facilitate a better understanding of the atherosclerosis process and lead to the development of future therapies. FAP is selectively expressed on activated VSMCs in the areas of active tissue remodeling, such as inflammatory lesions or neoplastic proliferation, but it is not expressed in resting fibroblasts in normal tissues. This is an intriguing molecule that is well suited to play a major role as a marker and target of activated myofibroblasts in atherosclerosis.

Data using FAP-targeted positron emission tomography and single-photon emission computed tomography in patients with acute myocardial infarction showed that the FAP expression exceeds the infarct region. This suggests that the role of FAP may include both replacement fibrosis in the scar and reactive fibrosis in the noninfarcted myocardium [16,28,29]. Furthermore, besides its potential role as a diagnostic marker [30,31], FAP appears to have a potential role as a therapeutic target in animal studies. It has been shown that genetic deletion of FAP leads to thicker scar formation and less ventricle dilatation following myocardial infarction [32], and immunomodulation with removal of FAP-expressing cells leads to the reversal of cardiac fibrosis [33]. In addition, FAP inhibition has been shown to increase angiogenesis in the peri-infarct zone, promoting ECM formation and possibly limiting scar expansion [34]. In our study, FAP staining was absent in coronary sections without atherosclerosis and increasingly present with increased plaque size and complexity, suggesting that FAP is a marker of activated myofibroblasts in human native atherosclerosis. VSMCs express different phenotypes during differentiation and proliferation and play a distinct role in native coronary artery disease, accelerated atherosclerosis after bypass surgery, and cardiac allograph vasculopathy. The more diffuse pattern of FAP staining visualized in our comparative analysis in bypass atherosclerosis and especially in post-transplant arteriosclerosis compared with native coronary atherosclerosis is likely related to the different pathophysiologies and morphologies of coronary stenoses in these patients. Vein graft atherosclerotic lesions are concentric and more diffuse, with a frequently absent fibrous cap. VSMC proliferation causes remodeling and arterialization of the vein with subsequent development in critical stenosis [35]. A study analyzing calcium scores in post bypass atherosclerosis has shown nonrapid progression of calcium scores, suggesting greater progression of the noncalcified plaque constituents [36].

The highly positive and more diffuse staining in post-heart transplant coronary arteriosclerosis, which in our study involved both the tunica intima and tunica media, may be explained by the multifactorial pathogenesis of cardiac allograph vasculopathy. It is postulated that initial injury to the endothelium (likely through an immunologic mechanism) stimulates the migration of activated VSMCs from the tunica media into the intima and also triggers proliferation, which results in luminal narrowing [37,38]. The above data support the presence of FAP in different forms of atherosclerosis and the role of FAP as a potential target, not only in native atherosclerosis, but also in vein graft atherosclerosis and post-transplant arteriosclerosis, which currently have very limited therapeutic options available. With further development of specific FAP inhibitors, a possible role of FAP as a diagnostic marker and potential target for interventions in different forms of atherosclerosis will be further determined.

## 5. Conclusions

Immunohistochemical localization of FAP in different types of human atherosclerotic lesions showed the presence of FAP expression with different patterns of staining in advanced atherosclerotic plaque, vein graft atherosclerosis lesions, and arteriosclerosis after a heart transplant. FAP may be a potential diagnostic marker and target for interventions, not only in coronary atherosclerotic plaque, but also in other forms of CAD, which currently have limited treatment options.

## Data Availability

Data are contained within the article.
